# Laveran et l'Académie nationale de médecine

**DOI:** 10.48327/mtsi.v3i1.2023.327

**Published:** 2023-03-01

**Authors:** Yves Buisson

**Affiliations:** Membre de l'Académie nationale de médecine; SFMTSI Société francophone de médecine tropicale et santé internationale (ancienne SPE), Hôpital Pitié-Salpêtrière, Pavillon Laveran, 47-83 Boulevard de l'Hôpital, 75651 Paris cedex 13, France; * Actes du Colloque – Centenaire de la mort d'Alphonse Laveran. 24 novembre 2022, Paris / Proceedings of the Conference – Centenary of the death of Alphonse Laveran. 24 November 2022, Paris

**Keywords:** Alphonse Laveran, Académie de médecine, Paludisme, Hématozoaire, Alphonse Laveran, Academy of Medicine, Malaria, Haematozoon

## Abstract

En novembre 1880, Alphonse Laveran, en poste à l'hôpital militaire de Constantine, adresse à l'Académie de médecine une « Note sur un nouveau parasite trouvé dans le sang de plusieurs malades atteints de fièvre palustre ». Léon Colin, professeur au Val-de-Grâce, en est le rapporteur, mais il n'est pas convaincu par ces observations, ni par les deux notes complémentaires envoyées par Laveran en décembre 1880 et en octobre 1881, scepticisme partagé par d'autres académiciens tels que Joseph Laboulbène et Émile Duclaux. Douze années seront nécessaires à Laveran pour surmonter l'incrédulité de la communauté scientifique française. Trois ouvrages fondamentaux offerts à l'Académie témoignent de la ténacité avec laquelle il a progressivement réussi à convaincre la majorité de ses collègues: le *Traité des fièvres palustres avec la description des microbes du paludisme* en 1884, *Des hématozoaires du paludisme* en 1887, et *Du paludisme et de son hématozoaire* en 1891.

Laveran est élu à l'Académie de médecine le 26 décembre 1893. Sa démission du Corps de santé militaire lui permet de participer assidûment aux séances et d'intervenir dans les débats concernant les maladies infectieuses et tropicales, l'hygiène et la prophylaxie.

En 1907, l'obtention du prix Nobel pour ses recherches sur le paludisme, les trypanosomiases et les maladies coloniales, vient couronner ses travaux tout en honorant l'Académie. Laveran en est élu vice-président pour l'année 1919 et président pour 1920, année du centenaire de l'Académie dont il organise la célébration au détriment de sa santé. Il meurt deux ans plus tard, ayant accompli son devoir jusqu'au bout de ses forces.

## Abstract (en)

In November 1880, Alphonse Laveran, stationed at the Constantine military hospital, addressed to the Academy of Medicine a “Note on a new parasite found in the blood of several patients with malaria fever”. Léon Colin, professor at the Val-de-Grâce school, is the rapporteur, but he is not convinced by these observations, nor by two additional notes sent by Laveran in December 1880 and October 1881. This skepticism is shared by other academicians such as Joseph Laboulbène and Émile Duclaux.

Twelve years will be necessary for Laveran to overcome the disbelief of the French scientific community. Three fundamental books donated to the Academy testify to the tenacity with which he gradually succeeded in convincing most of his colleagues: *Traité des fièvres palustres avec la description des microbes du paludisme* in 1884, *Des hématozoaires du paludisme* in 1887, and *Du paludisme et de son hématozoaire* in 1891.

Laveran was elected to the Academy of Medicine on December 26, 1893. His resignation from the Military Health Corps enabled him to participate assiduously in meetings and to intervene in debates concerning infectious and tropical diseases, hygiene and prophylaxis. Obtaining the Nobel Prize in 1907 for his work on malaria, trypanosomiasis and colonial diseases crowned his work while honoring the Academy. Laveran was elected vice-president for the year 1919 and president for 1920, the year of the centenary of the Academy, the celebration of which he organized to the detriment of his health. He died two years later, having fulfilled his duty to the end of his strength.

## Introduction

**Figure 1:**
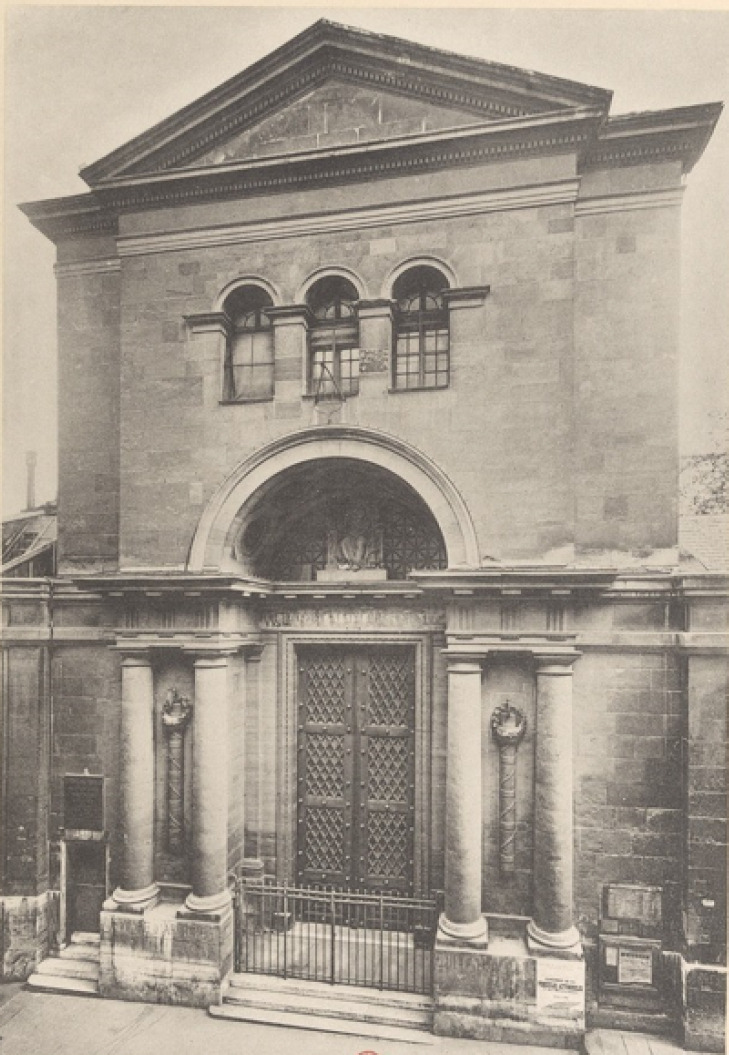
Siège de l'Académie de médecine en 1893 (rue des Saints-Pères, Chapelle de l'ancien hôpital de la Charité) (source: Bibliothèque de l'Académie nationale de médecine) Seat of the Academy of Medicine in 1893 (rue des Saints-Pères, chapel of the former Charity hospital, Paris) (source: Library of the National Academy of Medicine)

Lorsque Alphonse Laveran est élu membre de l'Académie nationale de médecine, le 26 décembre 1893, l'institution siège au 49 rue des Saints-Pères. Depuis 1850, elle occupe la chapelle de l'ancien hôpital de la Charité (Fig. 1). Elle s'installera le 25 novembre 1902 dans ses locaux actuels de la rue Bonaparte. En 1893, l'Académie de médecine est composée de 100 membres titulaires répartis en 12 sections: 1) anatomie et physiologie, 2) pathologie médicale, 3) pathologie chirurgicale, 4) thérapeutique et histoire naturelle, 5) médecine opératoire, 6) anatomie pathologique, 7) accouchements, 8) hygiène publique, médecine légale et police médicale, 9) médecine vétérinaire, 10) physique et chimie médicales, 11) pharmacie et 12) membres associés libres. Âgé de 48 ans, Laveran est alors titulaire de la Chaire d'hygiène militaire et de médecine légale à l’École d'application du Val-de-Grâce. Après des années de persévérance pour surmonter le scepticisme affiché vis-à-vis de sa découverte, il fait preuve d'opiniâtreté et présente, pour la cinquième fois en 2 ans, sa candidature pour une place vacante dans la section « Thérapeutique et histoire naturelle ». Il l'emporte avec 59 voix sur 79 votants, devançant Henri Huchard et Raphaël Blanchard qui, plus tard, le rejoindront sur les sièges de l'Académie.

En 1895, Laveran est élu à l'Académie des sciences comme membre correspondant, puis membre titulaire en 1901 au terme d'une compétition serrée avec Charles Richet. Tous deux recevront le prix Nobel de physiologie ou médecine, Laveran en 1907 pour ses travaux sur le paludisme, les trypanosomiases et les maladies coloniales, Richet en 1913 pour la découverte de l'anaphylaxie.

Si une telle pluie d'honneurs couronne l’œuvre féconde de Laveran, elle récompense aussi sa ténacité et sa volonté inflexible de convaincre un monde scientifique désespérément incrédule devant une découverte majeure.

## La Longue Marche Vers La Reconnaissance

**Figure 2:**
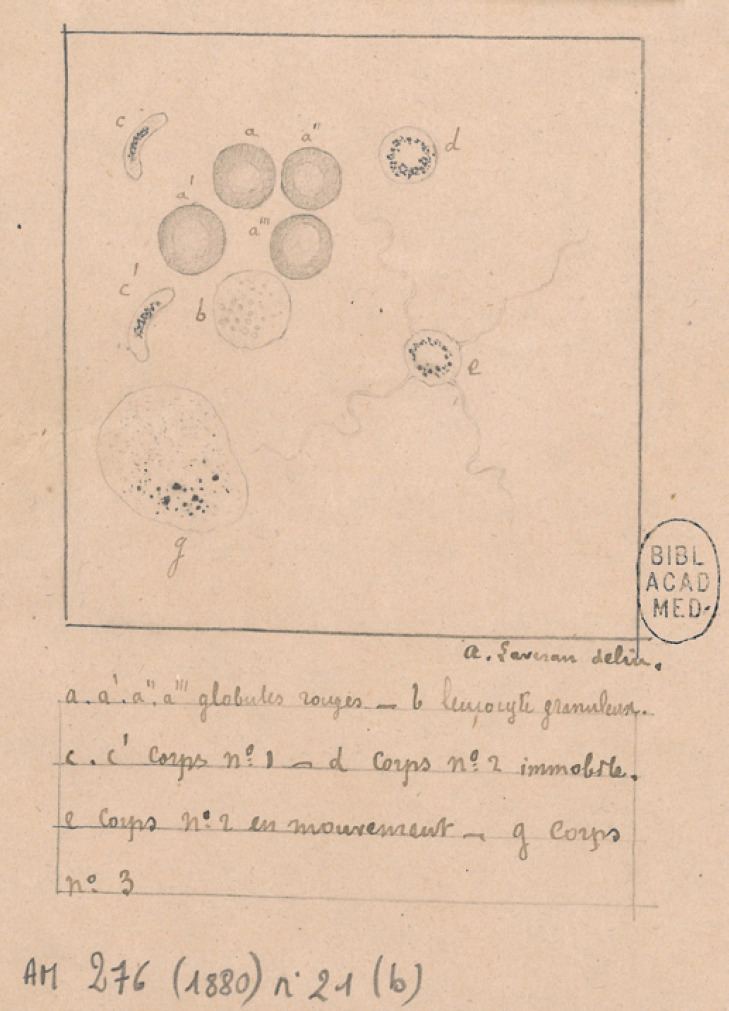
Note manuscrite du 11 novembre 1880 adressée par Laveran à l'Académie de médecine avec la première description des Corps n°1 (allongés, en croissant), des Corps n°2 (arrondis, avec ou sans exflagellation) et des Corps n° 3 (amœboïdes) (source: Bibliothèque de l'Académie nationale de médecine) Handwritten note addressed by Laveran to the Academy of Medicine with the first description of Bodies No. 1 (elongated, crescent-shaped), Bodies No. 2 (round, with or without exflagellation) and Bodies No. 3 (amoeboids) (source: Library of the National Academy of Medicine)

Ses premières observations avaient été adressées à l'Académie de médecine. Il était alors professeur agrégé du Val-de-Grâce, médecin-major de 1^re^ classe en poste à l'hôpital militaire de Constantine. Il s’était naturellement confié à Léon Colin, médecin militaire de 15 ans son aîné, professeur au Val-de-Grâce, élu depuis quelques mois à l'Académie, pour qu'il présente devant la Compagnie une « Note sur un nouveau parasite trouvé dans le sang de plusieurs malades atteints de fièvre palustre » le 23 novembre 1880. Laveran y décrivait des éléments parasitaires provisoirement nommés Corps n^os^ 1, 2 et 3 à l'appui d'un schéma détaillé (Fig. 2). Mais Colin, malgré une bienveillance de principe pour son jeune confrère, avait d'autres idées sur les fièvres intermittentes qu'il attribuait à « une intoxication tellurique » [3] et relevait « la difficulté de concilier la nature animée du germe avec l'absence de contagiosité de l'affection ». De plus, il ne cachait pas son scepticisme sur le caractère pathologique des corpuscules décrits par Laveran, évoquant la possibilité de leucocytes mélanifères. Lors de la séance du 28 décembre 1880, Colin présentait, sans conviction, la « Deuxième note relative à un nouveau parasite trouvé dans le sang des malades atteints de fièvres palustres » où Laveran réfutait l'hypothèse des leucocytes mélanifères par une description morphologique soigneuse des parasites observés. En présentant la « Troisième note relative aux éléments parasitaires trouvés dans le sang des malades atteints d'impaludisme » le 25 octobre 1881, Colin ne retenait qu'une seule phrase, pensant y trouver la confirmation de sa théorie « tellurique »; c’était une observation annexe faite par Laveran dans les flaques d'eau d'une localité insalubre, à savoir « des éléments composés d'une petite masse transparente, douée de mouvements amiboïdes et renfermant des grains pigmentés qui rappelaient ceux qu'on observe dans le sang des individus impaludés. Ces êtres très simples représentent peut-être la forme sous laquelle les parasites de l'impaludisme existent en dehors du corps humain ».

La Commission nommée pour analyser ces trois notes a prudemment choisi d'attendre de nouvelles données pour se prononcer. Cette indécision était alors partagée par d'autres académiciens, notamment par l'entomologiste Joseph Laboulbène, perplexe devant ce parasite inconnu dont on ignorait les voies de pénétration chez l'homme et par le Pasteurien Émile Duclaux qui, refusant d'admettre que le dogme de l’étiologie microbienne des maladies infectieuses fût bousculé, considérait que Laveran n'avait pas compris « la signification des faits lui passant sous les yeux » [4].

En 1884, nommé titulaire de la Chaire d'hygiène militaire à l’École du Val-de-Grâce, Laveran est rappelé à Paris après 5 années passées en Algérie. Il rassemble les observations qu'il y a effectuées dans un recueil fondamental de 548 pages, le *Traité des fièvres palustres avec la description des microbes du paludisme* dans lequel il postule que ce microbe se trouve à l’état de parasite chez les moustiques. Probablement séduit par cette hypothèse, c'est Laboulbène qui présente et offre à l'Académie la première édition de cet ouvrage de référence [5]. Bien plus tard, en prononçant l’éloge posthume de Laveran, Charles Achard regrettera « que ce savant, occupé à cette époque par les lourdes fonctions de professeur, n'ait pu avoir la possibilité de se rendre en Algérie pour démontrer par l'expérience ce que Ronald Ross a élucidé aux Indes en 1895 ».

Pourtant, la découverte de Laveran demeure confidentielle. Elle est totalement ignorée par Raphaël Blanchard, auteur d'un imposant traité de zoologie médicale dont le premier volume, rédigé en 1886, incluait l’étude des protozoaires [1]. Mais Laveran n'est pas rancunier; il sera le rapporteur du dossier de candidature de Blanchard à l'Académie de médecine et lui permettra d'y être élu en 1894. Devenus amis, ils publieront ensemble *Les hématozoaires de l'homme et des animaux* en 1895 [8].

Le 26 juillet 1887, c'est un célèbre chirurgien, le baron Hippolyte Larrey, qui apporte son soutien à Laveran en présentant à l'Académie son mémoire intitulé *Des hématozoaires du paludisme.* Il conclut: « Enfin, M. Laveran a non seulement confirmé, par lui-même, en France comme en Algérie et en Italie, mais encore il a fait confirmer, par d'autres observateurs, le fait, acquis désormais, de l'existence des hématozoaires du paludisme. »

Le 21 avril 1891, Laboulbène offre à l'Académie l'ouvrage de Laveran intitulé *Du paludisme et de son hématozoaire* dans lequel les descriptions parasitologiques et histologiques sont complétées par des données prophylactiques et thérapeutiques dont la rigueur scientifique semble avoir vaincu ses derniers doutes [6].

## Laveran Académicien

Quelques mois après son élection à l'Académie de médecine, la situation militaire de Laveran va l’éloigner de Paris. Ayant achevé son temps de professorat au Val-de-Grâce en 1894, il sollicite en vain sa nomination à la Chefferie de l'hôpital militaire de Vincennes pour pouvoir assister aux séances hebdomadaires de l'Académie. Il est affecté à Lille comme médecin chef de la Place et de l'hôpital militaire, puis muté à Nantes comme directeur du service de santé du 11^e^ Corps d'armée. L'année suivante, il demande sa mutation à Orléans comme directeur du service de santé du 5^e^ Corps d'armée, toujours motivée par l'intérêt de sa présence à l'Académie. Le ministre lui répond sèchement qu'il n'est pas le seul médecin militaire à faire partie de l'Académie, il y a déjà Léon Colin, Émile Vallin, Louis Kelsch, Jules Chauvel et Jean Marty… La réaction de Laveran est immédiate; il présente sa démission du Service de santé des armées le 15 décembre 1896, à l’âge de 51 ans.

De retour à Paris, ayant intégré l'Institut Pasteur comme chercheur bénévole avec le titre de chef de service honoraire, Laveran se révèle un académicien assidu, dynamique et polyvalent. Il monte régulièrement à la tribune pour intervenir avec l'autorité d'un expert dans les principaux débats concernant les maladies infectieuses et tropicales, l'hygiène et la prophylaxie. Le paludisme restant l'une de ses principales préoccupations, il fait connaître l'emploi préventif de la quinine en 1896, le rôle de la rate dans le paludisme en 1897, et rapporte en 1899 le travail de Ronald Ross dans sa « Note pour l'histoire du parasite du paludisme en dehors de l'organisme humain ». Il s'intéresse aussi au paludisme autochtone, encore très répandu en France. Dans son rapport sur l’étude de Louis-Henri Roché décrivant l'extinction du paludisme en Puisaye [11], il souligne l'importance du drainage du sol, de l'assèchement des marais et des étangs, et montre qu'une politique de grands travaux peut être efficace pour faire reculer l'endémie palustre dans une région. En 1900, il propose la création d'une commission du paludisme, principalement destinée aux colonies, les foyers palustres de métropole se réduisant de plus en plus. Cette commission rédige un projet d'instruction pour la prophylaxie du paludisme [10] dont les recommandations englobent le traitement du milieu (eaux stagnantes et moustiques) et le traitement de l'homme par la quinine. Laveran interviendra encore à plusieurs reprises au sujet de la lutte contre le paludisme en Corse, à Madagascar, en Algérie et dans les Balkans.

Mais le paludisme n'est pas la seule maladie tropicale qui intéresse Laveran. Plusieurs de ses communications concernent la pathologie exotique, en particulier la maladie du sommeil, l'amibiase, les leishmanioses et les mycétomes. À partir de 1900, il présente les travaux effectués à l'Institut Pasteur en collaboration avec Félix Mesnil sur les trypanosomiases humaines et animales (nagana, surra) qui constitueront la trame de l'ouvrage *Trypanosomes et trypanosomiases* présenté en 1912 à l'Académie [9].

**Figure 3:**
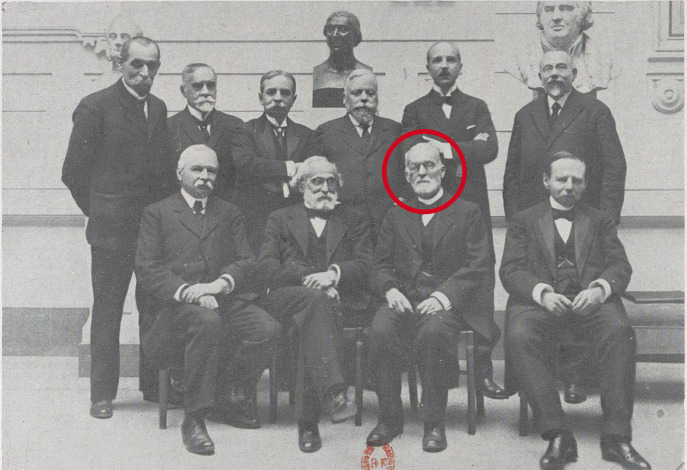
Laveran et les membres de la section « Thérapeutique et histoire naturelle » en 1919 (source: Bibliothèque de l'Académie nationale de médecine) Laveran and his fellow members of the “Therapeutics and natural history” section in 1919 (source: Library of the National Academy of Medicine)

C'est aussi comme militaire et hygiéniste qu'il intervient sur des sujets aussi variés que la pathogénie du coup de chaleur, la pathogénie de l'appendicite, la prophylaxie de la tuberculose, la purification de l'eau de boisson du soldat en campagne et les filtres Lapeyrère, la qualité microbiologique des eaux minérales, le fonctionnement défectueux du service des eaux à Paris, la désinfection, la dératisation et les vaccinations (Fig. 3). Au nom de la commission permanente des épidémies, il rédige un rapport général pour le président du Conseil sur les épidémies qui ont sévi en France pendant l'année 1899 [7].

## L'apogée

Premier lauréat français du prix Nobel de physiologie ou médecine, Laveran est le troisième membre de l'Académie de médecine honoré par le Karolinska Institutet de Stockholm après Marie Curie (prix Nobel de physique en 1903) et Henri Moissan (prix Nobel de chimie en 1906). Le 17 décembre 1907, le président de l'Académie, Armand Gautier, signale cette distinction par une déclaration d'une remarquable sobriété: « Je crois être l'interprète de vos sentiments en félicitant notre savant collègue, M. Laveran, pour le prix Nobel (Médecine) que vient de lui attribuer l'Institut Carolin de Stockholm. C'est une récompense bien méritée pour ses beaux travaux sur l'hématozoaire de la malaria, ses recherches sur la maladie du sommeil et en général sur les trypanosomiases et les maladies coloniales. C'est aussi un honneur qui rejaillit sur notre Académie et sur notre pays scientifique tout entier. Je prie donc, en votre nom, notre collègue M. Laveran d'en recevoir nos bien sincères compliments. » La réponse de Laveran, la semaine suivante, n'est pas moins laconique: « À mon grand regret, j’étais absent lorsque notre président m'a adressé, dans la dernière séance, des félicitations à l'occasion du prix Nobel de médecine. Je tiens à dire que j'ai été très touché de ces félicitations qui m’étaient adressées au nom de l'Académie de médecine, et à remercier M. le Président et tous mes collègues. »

**Figure 4:**
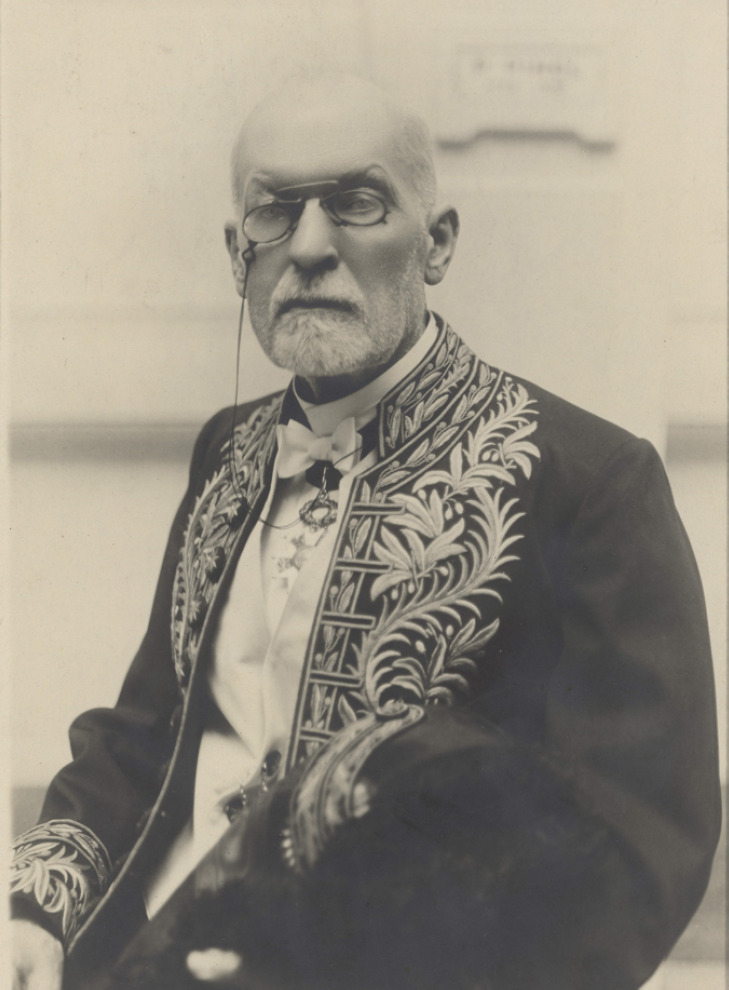
Laveran président de l'Académie de médecine en 1920 (source: Bibliothèque de l'Académie nationale de médecine) Alphonse Laveran, president of the Academy of Medicine in 1920 (source: Library of the National Academy of Medicine)

Membre du Conseil d'administration depuis 1910, Laveran est élu le 24 décembre 1918 vice-président de l'Académie de médecine pour l'année 1919, puis président pour l'année 1920 (Fig. 4). Il prend ainsi la succession d'un chirurgien militaire de renom, le médecin général inspecteur Edmond Delorme. Lors de sa première allocution, le 6 janvier 1920, il annonce: « L'Ordonnance royale qui a créé l'Académie de médecine est datée du 20 décembre 1820, le Centenaire de notre Académie tombera donc le 20 décembre prochain. Il y a eu parmi nous unanimité pour décider qu'il y avait lieu de célébrer cet anniversaire en rappelant les grands services rendus par notre Académie à la Science et à la Patrie. C'est avec une légitime fierté que nous évoquerons à cette occasion les grandes figures des plus illustres de nos prédécesseurs: Laënnec, Pinel, Dupuytren, Magendie, Velpeau, Trousseau, Orfila, Cruveilhier, Bouillaud, Malgaigne, Claude Bernard, Marey, Villemin, Pasteur, le plus grand de tous ! […] Je souhaite que l'année du Centenaire soit glorieuse pour l'Académie et que soient exaucés les vœux que nous avons émis à diverses reprises pour rendre plus efficace la lutte contre la tuberculose et l'alcoolisme. »

La célébration du centenaire dure trois jours. La première journée, ouverte le 20 décembre 1920 par le président de la République Alexandre Millerand, est consacrée à la séance solennelle d'inauguration en présence d'un grand nombre de personnalités civiles et militaires, françaises et étrangères. Laveran retrace l'historique de l'Académie de médecine et rappelle que, dès sa création, le Gouvernement avait pris « l'engagement moral de consulter l'Académie sur toutes les questions intéressant la santé publique » [2]. Les journées suivantes sont occupées par les conférences prononcées par Louis Vaillard (L'hygiène publique), Maurice Hanriot (Les bienfaiteurs de l'Académie), Anatole Chauffard (Un siècle de médecine), Lucien Camus (La vaccine), Gédéon Meillère (L'hydrologie) et Edmond Delorme (La chirurgie). La célébration s'achève par une réception à l'Hôtel de Ville et par un banquet d'adieu.

Lorsqu'il cède le fauteuil de président à son successeur Louis-Gustave Richelot le 4 janvier 1921, Laveran est épuisé. Il meurt l'année suivante, le 18 mai 1922. C'est Émile Brumpt qui annonce son décès aux membres de la Compagnie: « Les fêtes du Centenaire de l'Académie qu'il présida et à l'inauguration desquelles il prit une part si active, furent pour le professeur Laveran une cause de grande fatigue, et marquèrent le déclin de son activité physique. À dater de cette époque, il ne fréquenta plus avec la même régularité les sociétés qui s'honoraient de le compter parmi leurs membres. Tous ceux qui le connaissaient, et qui savaient l'intérêt qu'il prenait aux débats scientifiques, comprirent que ce changement profond dans ses habitudes indiquait une fin prochaine. […] Honorons-nous d'avoir pu le compter parmi nous et gardons de lui le souvenir d'un homme qui a grandement contribué à augmenter le prestige scientifique de son pays et que l'humanité a le devoir de considérer comme un de ses bienfaiteurs. »

Son éloge funèbre est prononcé le 10 décembre 1929 par Charles Achard, Secrétaire général de l'Académie de médecine. Il débute ainsi: « Découvrir la cause d'une maladie grave qui ravage une grande partie de notre globe et qui, mettant obstacle à la civilisation de ces régions, est pour l'humanité un fléau plus meurtrier que la peste et le choléra, n'est-ce pas un titre de gloire dont l’éclat l'emporte sur beaucoup d'autres renommées scientifiques? Non moins que par son labeur acharné, l'homme qui fit cette découverte fut, par la belle simplicité de sa vie et par la droiture impeccable de son caractère, une des plus nobles figures médicales qu'il soit donné au biographe de retracer et au panégyriste de proposer en exemple au respect de la postérité. »

Cette belle simplicité et son profond attachement à l'Académie sont toujours lisibles sur sa pierre tombale au cimetière Montparnasse. Savant de renommée internationale, couvert d'honneurs et de décorations, Laveran avait voulu que son épitaphe se limitât à ces mots: « Docteur A. Laveran, Membre de l'Institut et de l'Académie de médecine, 1845-1922 ».

## Liens D'intérêts

L'auteur ne déclare aucun lien d'intérêt.
